# Supplementary material for: Seminoma and Embryonal Carcinoma Footprints Identified by Analysis of Integrated Genome-Wide Epigenetic and Expression Profiles of Germ Cell Cancer Cell Lines
Source: PLoS One. 2014 Jun 2;9(6):e98330. doi: 10.1371/journal.pone.0098330 (PMC4041891; doi:10.1371/journal.pone.0098330)
Supplement: File S1 — ZIP file containing DMRforPairs output for significant regions. Please start from the html files. (ZIP) [file pone.0098330.s008.zip › figures/33800.pdf]

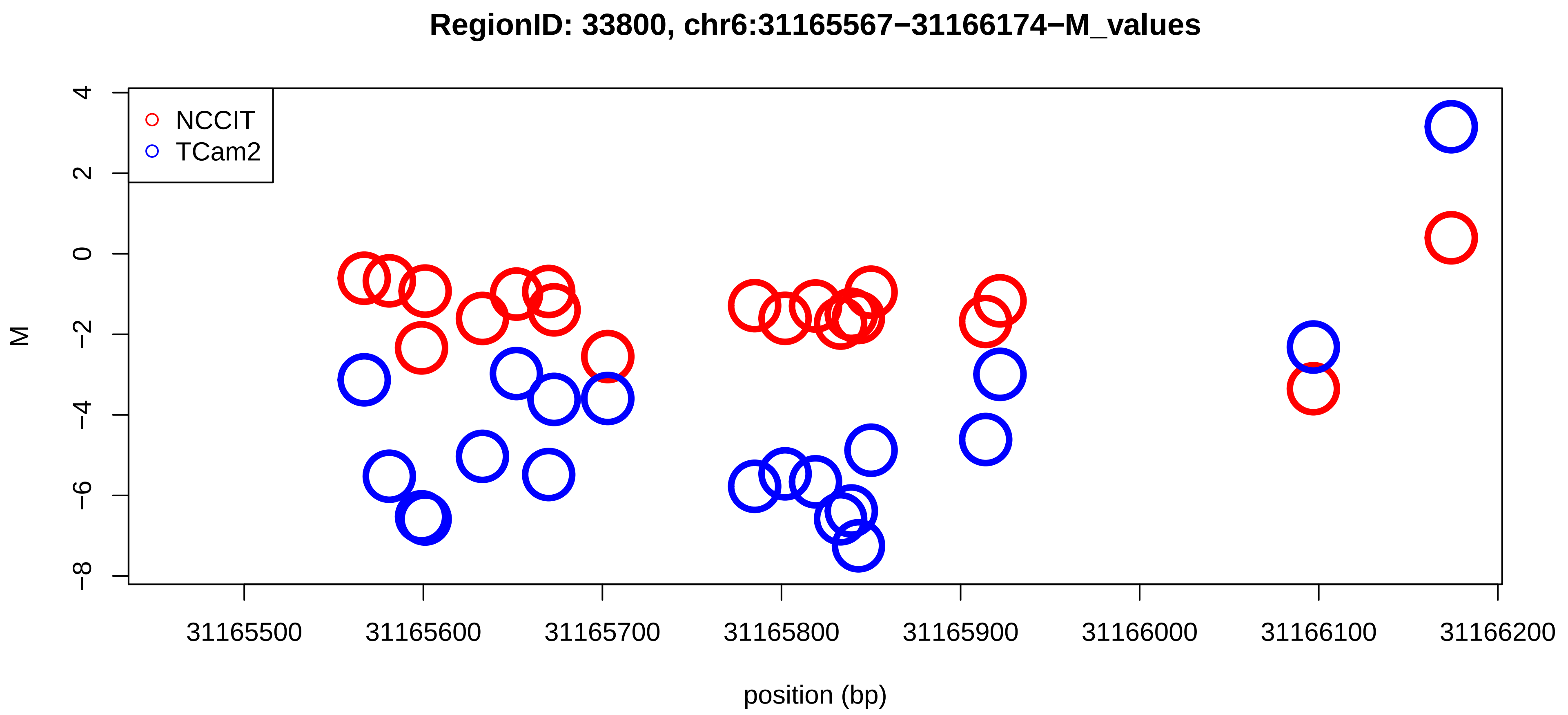
RegionID: 33800, chr6:31165567−31166174−M_values
NCCIT
TCam2
M
4
2
0
−2
−4
−6
−8
31165500
31165600
31165700
31165800
31165900
31166000
31166100
31166200
position (bp)

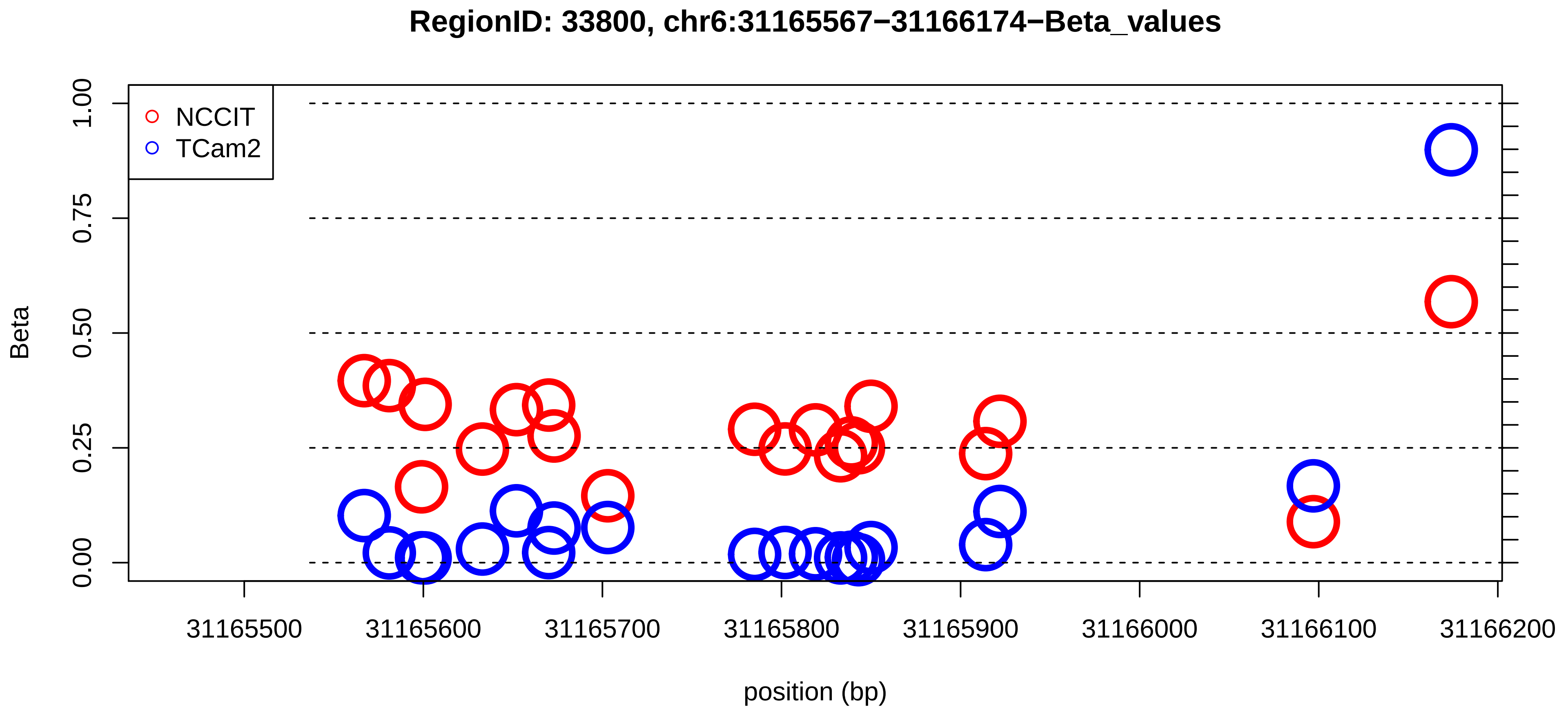
RegionID: 33800, chr6:31165567−31166174−Beta_values
NCCIT
TCam2
Beta
1.00
0.75
0.50
0.25
0.00
31165500
31165600
31165700
31165800
31165900
31166000
31166100
31166200
position (bp)

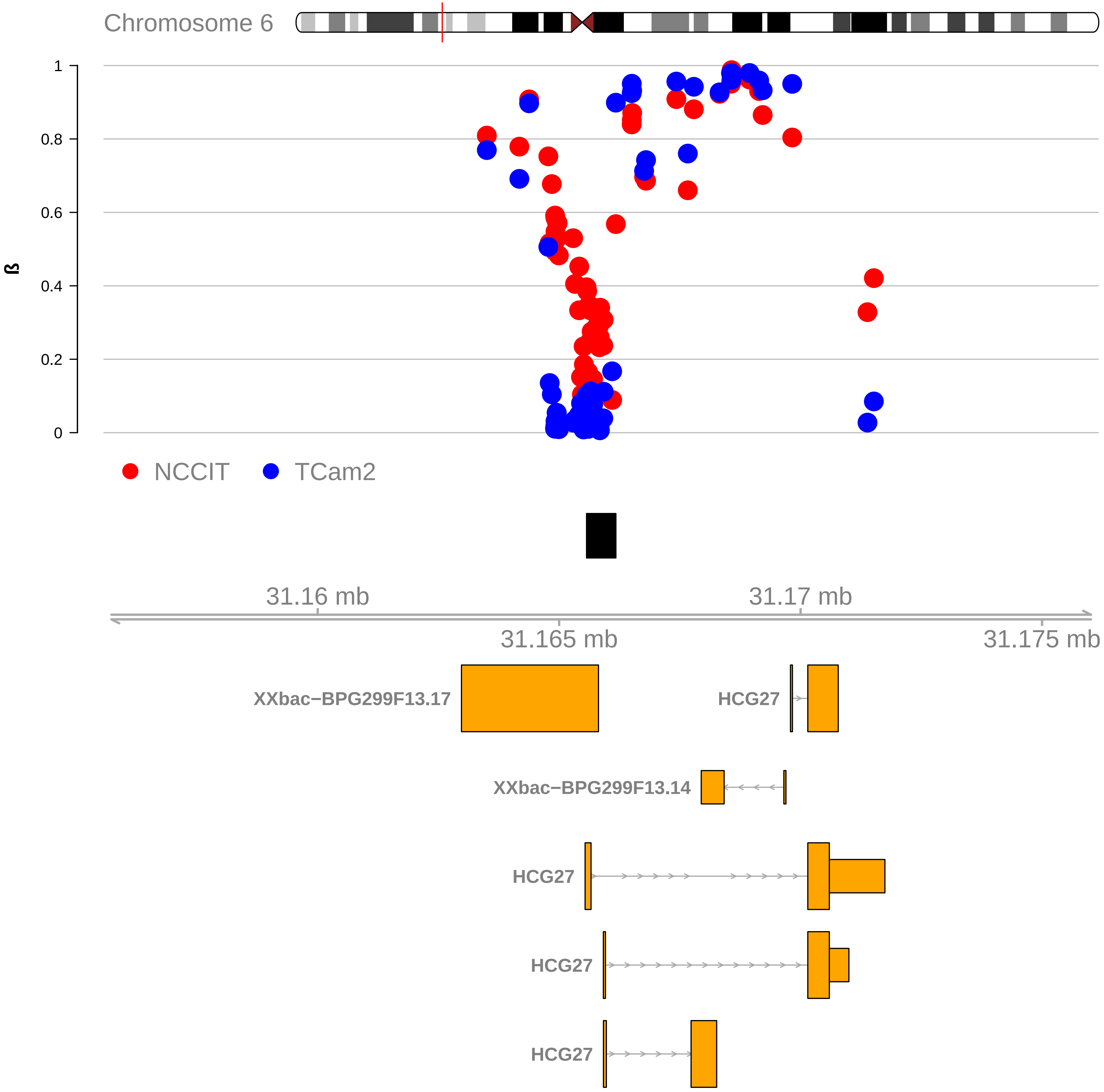

Chromosome 6
1
0.8
0.6
0.4
0.2
0
ß
NCCIT
TCam2
31.16 mb
31.17 mb
31.165 mb
31.175 mb
XXbac−BPG299F13.17
HCG27
XXbac−BPG299F13.14
HCG27
HCG27
HCG27
